# Inflammation Without Effective Immunity in Ovarian Cancer: From Early Translational Observations to Histotype-Dependent Immunometabolic Ecosystems

**DOI:** 10.3390/cancers18172782

**Published:** 2026-08-27

**Authors:** Manuela Neri, Paolo Albino Ferrari, Valerio Vallerino, Gabriele Sole, Antonio Macciò

**Affiliations:** 1 Department of Gynecological Oncology, Azienda di Rilievo Nazionale ed Alta Specializzazione “G. Brotzu”, 09121 Cagliari, Italy; manuela.neri@aob.it (M.N.); valerio.vallerino@aob.it (V.V.); antoniopm.maccio@aob.it (A.M.); 2 Department of Oncological Surgery, Azienda di Rilievo Nazionale ed Alta Specializzazione “G. Brotzu”, 09121 Cagliari, Italy; gabriele.sole@aob.it; 3 Department of Thoracic Surgery, Azienda di Rilievo Nazionale ed Alta Specializzazione “G. Brotzu”, 09121 Cagliari, Italy; 4 Department of Surgical Sciences, University of Cagliari, SS 554 km 4.500, 09042 Monserrato, Italy

**Keywords:** epithelial ovarian cancer, malignant ascites, tumor microenvironment, immunometabolism, IL-6, tumor-associated macrophages, ferroptosis, T-cell dysfunction, immune checkpoint inhibition, histotype

## Abstract

Epithelial ovarian cancer (EOC) comprises biologically and immunologically distinct histotypes and frequently develops within a chronically inflamed tumor microenvironment, particularly in advanced disease with malignant ascites. This narrative review revisits translational observations from the 1990s–2000s showing impaired lymphomonocyte proliferation, altered Fas/CD25 signaling, and abundant cytokine release in ovarian cancer effusions. These findings are interpreted as direct evidence of dysfunctional immune activation, but not as retrospective proof of T-cell exhaustion according to contemporary molecular, transcriptional, epigenetic, or functional definitions. Modern single-cell and spatial studies provide independent evidence that malignant ascites is a dynamic ecosystem containing heterogeneous T-cell and macrophage states and that immune architecture differs across anatomical compartments and histotypes. Within this framework, cytokine signaling, macrophage plasticity, iron/redox biology, ferroptosis susceptibility, adipocyte–tumor crosstalk, and systemic metabolic dysfunction are considered at different levels of evidentiary strength, with ovarian cancer-specific data distinguished from pan-cancer or preclinical extrapolation. Clinical experience with immune-checkpoint inhibitors further illustrates the distinction between immune-cell presence and effective immunity: single-agent activity has generally been modest, whereas the phase III ENGOT-ov65/KEYNOTE-B96 trial demonstrates that clinically meaningful benefit can emerge in an appropriate therapeutic and biomarker-selected context. We propose that “inflammation without effective immunity” is best viewed as an overarching, histotype- and context-dependent immunometabolic framework rather than a uniform ovarian cancer phenotype. The concept remains hypothesis-generating and requires prospective validation before biomarker or therapeutic implementation.

## Simple Summary

Ovarian cancer often develops in a chronically inflamed microenvironment, particularly in patients with malignant ascites, yet inflammation does not necessarily generate effective antitumor immunity. This review revisits early translational studies showing that lymphomononuclear cells recovered from ovarian cancer effusions could display impaired proliferation and altered activation despite abundant inflammatory cytokines. These historical findings are distinguished from, rather than equated with, modern molecular definitions of T-cell exhaustion and other immune states. Contemporary single-cell and spatial studies now demonstrate marked heterogeneity of T cells, macrophages, stromal cells, and their interactions across ascites, primary tumors, metastases, and ovarian cancer histotypes. We therefore propose “inflammation without effective immunity” as a hypothesis-generating framework whose biological expression is histotype- and context-dependent. Its potential translational value lies in integrating inflammatory, immune, metabolic, and ascites-derived biomarkers and in identifying rational therapeutic combinations, but it should not yet be regarded as established clinical guidance.

## 1. Introduction

Epithelial ovarian cancer (EOC) continues to represent one of the most challenging malignancies in gynecologic oncology. Despite significant improvements in surgical techniques, perioperative management, molecular characterization, and maintenance therapies, the majority of patients with advanced-stage disease eventually experience recurrence and die from progressive cancer [1,2]. Over the last decade, considerable efforts have been directed toward understanding the interactions between ovarian cancer and the immune system. The presence of tumor-infiltrating lymphocytes, the prognostic significance of immune signatures, and the development of immune checkpoint inhibitors have all highlighted the potential importance of antitumor immunity in ovarian cancer [3]. Nevertheless, clinical responses to immunotherapy remain substantially lower than those observed in malignancies such as melanoma, non-small-cell lung cancer, or renal cell carcinoma [4,5,6,7,8].

This discrepancy suggests that ovarian cancer may not simply be characterized by insufficient immune recognition. Instead, the ovarian tumor microenvironment may contain abundant immune populations that are unable to generate effective and durable antitumor responses. Recent advances have introduced several concepts that help explain this phenomenon, including immune exhaustion, chronic inflammatory activation, macrophage polarization, metabolic competition, ferro-metabolic signaling, and immunometabolic reprogramming [3,9]. These mechanisms are increasingly recognized as interconnected components of a complex biological network that regulates tumor progression and therapeutic resistance.

Several biological roots of this framework can be traced to translational studies performed during the 1990s and early 2000s on malignant ascites and neoplastic effusions from ovarian cancer patients [10,11]. Although the language of modern immuno-oncology was not yet available, those investigations identified abnormalities involving immune-cell activation, cytokine signaling, macrophage biology, iron metabolism, and systemic inflammatory responses [11,12,13]. The central hypothesis of this review is that ovarian cancer may represent a condition of inflammation without effective immunity. In this model, chronic inflammatory activation coexists with impairment of immune competence, generating a tumor-promoting immunometabolic ecosystem that favors progression, cachexia, and resistance to therapy [10].

Importantly, epithelial ovarian cancer should not be treated as a single immunological entity. High-grade serous, clear-cell, endometrioid, mucinous, and low-grade serous carcinomas arise from distinct molecular backgrounds and display different patterns of immune infiltration, checkpoint expression, stromal organization, and macrophage composition. Spatially resolved profiling across 254 ovarian cancers has confirmed subtype-specific immune architectures and immunoregulatory targets, including distinct patterns in high-grade serous, clear-cell, mucinous, endometrioid, and low-grade serous carcinomas. Accordingly, “inflammation without effective immunity” is proposed here as an overarching framework that may arise through distinct histotype- and context-dependent immunometabolic configurations rather than as a uniform phenotype shared by all ovarian cancers [14,15].

## 2. Narrative Scope and Interpretive Framework

This manuscript is presented as a narrative and interpretive review rather than as a systematic review or meta-analysis. The synthesis is based on the peer-reviewed literature cited in this manuscript, including historical translational studies on ovarian cancer ascites and tumor-associated lymphomonocytes, contemporary reviews and mechanistic studies on cancer immunometabolism, and selected clinical or translational literature addressing immunotherapy resistance, macrophage biology, iron metabolism, ferroptosis, cachexia, and PARP biology.

The purpose of the review is not to claim that early studies directly demonstrated modern molecular entities such as T-cell exhaustion, macrophage polarization, or ferroptosis in their current definitions. Rather, the objective is to re-examine functional and inflammatory observations from ovarian cancer effusions through contemporary biological frameworks. A strict distinction is therefore maintained throughout this review between (i) findings directly demonstrated in historical ovarian cancer samples; (ii) mechanisms subsequently demonstrated in ovarian cancer using contemporary molecular or spatial approaches; and (iii) mechanistic interpretations extrapolated from broader cancer or preclinical literature. Terms such as exhaustion-like dysfunction and ferro-metabolic signaling are used only at the interpretive level unless the cited study directly measured the defining molecular, transcriptional, epigenetic, or functional features.

## 3. A Historical Hypothesis Revisited

The development of modern immuno-oncology has profoundly changed our understanding of cancer biology. Concepts such as T-cell exhaustion, immune checkpoint regulation, metabolic reprogramming, and macrophage polarization now occupy a central role in explaining tumor progression and resistance to therapy. However, scientific paradigms rarely emerge suddenly. In many cases, biological observations precede the conceptual frameworks required to interpret them [16,17,18,19,20,21]. This phenomenon appears particularly evident in ovarian cancer.

Long before the introduction of immune checkpoint inhibitors and before the emergence of the term “immunometabolism”, translational studies performed on ovarian cancer ascites had identified a striking biological paradox. Immune cells recovered from neoplastic effusions were surrounded by a highly inflammatory milieu rich in cytokines and activation signals, yet they displayed functional impairment [10,11,12]. At that time, these findings were interpreted primarily within the context of cytokine biology and tumor-induced immunosuppression. Today, however, they can be re-examined through a broader framework integrating inflammation, metabolism, immune dysfunction, and host adaptation. Viewed retrospectively, malignant ascites represented an in vivo biological compartment in which several mechanisms currently included within immunometabolic models were functionally suggested, although not molecularly defined [11]. Rather than isolated observations, these findings may represent early elements of a biological continuum linking chronic inflammation, metabolic adaptation, macrophage reprogramming, immune dysfunction, and therapeutic resistance [8,9,10].

## 4. Early Translational Evidence of Immune Dysfunction in Ovarian Cancer

One of the earliest indications that ovarian cancer might induce profound local immune dysregulation emerged from studies investigating tumor-associated lymphomonocytes isolated from malignant effusions (TALMs). These investigations demonstrated that TALMs exhibited markedly impaired proliferative responses when compared with autologous peripheral blood mononuclear cells [13]. The impairment persisted despite exposure to an inflammatory microenvironment characterized by abundant cytokine production and apparent immune activation. Cell-cycle analyses revealed accumulation within the G0/G1 phase of the cell cycle, indicating quiescent or early gap-phase arrest, suggesting an inability to progress toward effective immune activation and proliferation. Functionally, these findings indicated immune paralysis rather than simple immune absence [13].

Particularly relevant was the observation that this proliferative defect coexisted with elevated concentrations of multiple inflammatory mediators, including IL-6, TNF-α, IL-10, IFN-γ, and soluble IL-2 receptor [10,11]. Such coexistence of activation signals and functional impairment constitutes a central feature of dysfunctional immune activation. From a contemporary perspective, these findings resemble aspects of exhaustion-like immune phenotypes, because immune cells remain present within the tumor microenvironment and retain evidence of activation, yet fail to generate effective antitumor responses [19,20,21]. This interpretation should remain cautious, because the original studies were not designed to test modern molecular definitions of T-cell exhaustion.

Additional investigations further strengthened this hypothesis. Elevated circulating levels of inflammatory cytokines and soluble IL-2 receptor correlated with impaired T-cell responsiveness and markers of systemic inflammation [11]. These observations suggested that immune dysfunction was not restricted to the local tumor environment but reflected a broader host response involving both immune and metabolic compartments. The analysis of Fas receptor/CD95 and CD25/interleukin-2 receptor alpha-chain signaling pathways provided further insight into this paradox. Increased expression of activation markers coexisted with defective immune-cell function, suggesting that chronic stimulation may promote dysfunction rather than effective activation [12]. Collectively, these studies established a conceptual foundation for understanding ovarian cancer as a disease characterized not by immune silence, but by biologically ineffective immunity [10]. Table 1 summarizes the historical evidence of inflammatory and immunological processes that establish in advanced ovarian cancer and are associated with systemic disease.

## 5. The Ascitic Microenvironment as an Immunometabolic Niche

Malignant ascites represents one of the most distinctive biological features of advanced epithelial ovarian cancer. Traditionally considered a consequence of peritoneal dissemination and altered vascular permeability, ascites is now recognized as a highly active biological compartment capable of profoundly influencing tumor behavior, host responses, and therapeutic outcomes [33,34,35]. Rather than functioning as a passive reservoir of tumor cells, malignant ascites constitutes a dynamic ecosystem composed of neoplastic cells, tumor-associated macrophages (TAMs), lymphocytes, mesothelial cells, adipocytes, fibroblasts, extracellular vesicles, cytokines, chemokines, growth factors, and metabolic mediators [10,33,34,35].

**Figure 1:**
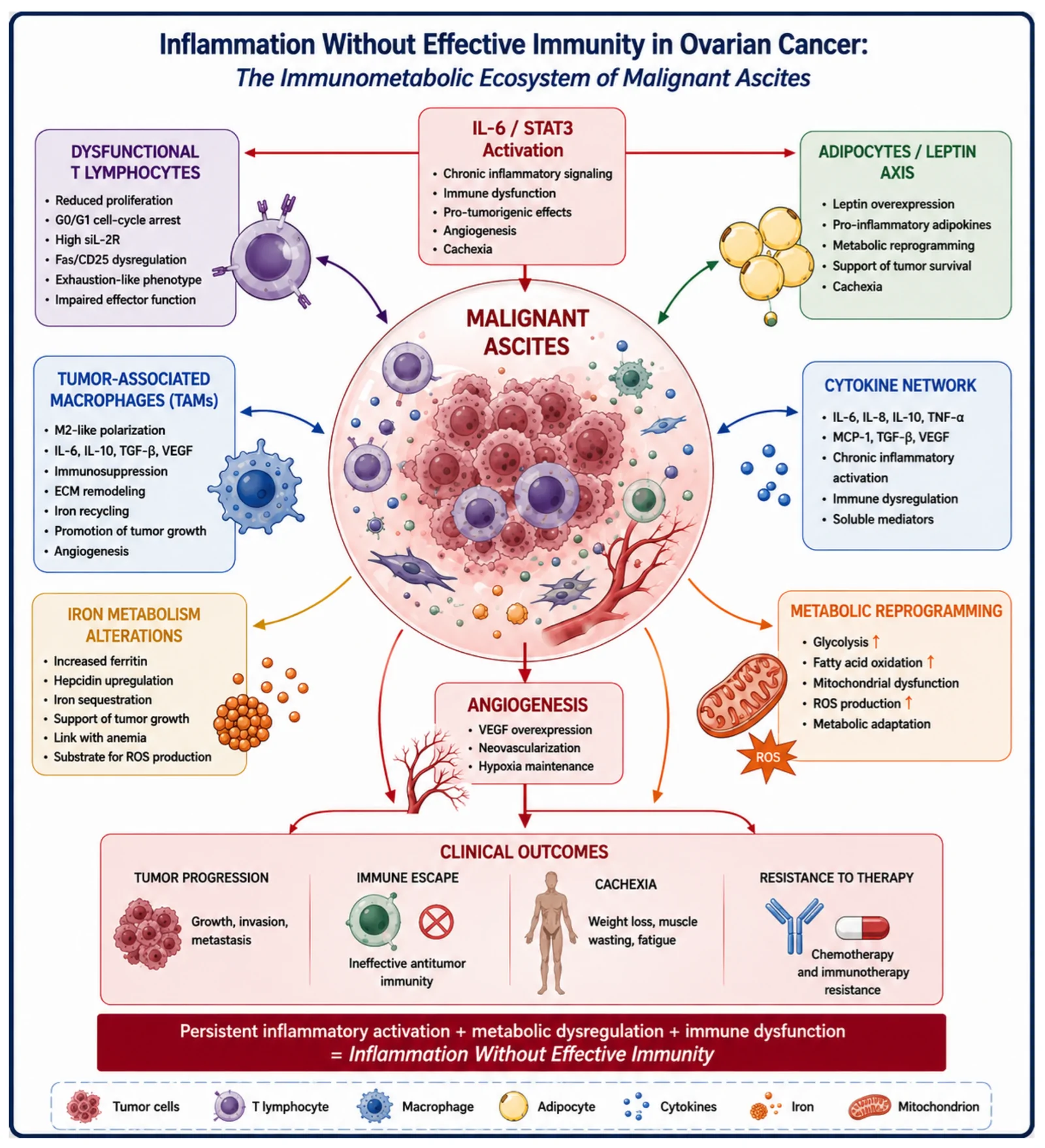
Malignant ascites as an immunometabolic ecosystem. Tumor cells, TAMs, T cells, adipocytes, cytokines, hypoxia, lactate, iron handling, mitochondrial stress, and ferroptotic susceptibility interact bidirectionally to sustain progression and therapeutic resistance.

Continuous interactions among these components generate a complex network that simultaneously promotes tumor progression and suppresses effective antitumor immunity. The biological relevance of ascites became evident through early translational studies demonstrating elevated concentrations of inflammatory mediators, particularly IL-6, TNF-α, IL-10, and soluble IL-2 receptor [10,11]. These observations suggested that ovarian cancer develops within a state of persistent inflammatory activation extending beyond the tumor itself. Subsequent investigations progressively expanded this inflammatory model toward a broader immunometabolic framework. The ascitic compartment emerged as a site where immune regulation, metabolic adaptation, angiogenesis, and host systemic responses converge [33,34,35] (Figure 1).

Contemporary single-cell and spatial profiling have now provided direct support for this ecosystem view. These approaches demonstrate that malignant ascites and metastatic ovarian cancer contain heterogeneous tumor, T-cell, myeloid, fibroblast, and stromal populations linked by site-specific cell–cell communication networks. Recent spatial studies further show that immune organization differs not only among histotypes but also among anatomical compartments within high-grade serous ovarian cancer. These data strengthen the interpretation of ascites as an active tumor–host interface while also cautioning against extrapolating one immune configuration to all ovarian cancers [14,15,37]. Independent ascites-focused single-cell studies identify heterogeneous macrophages and ligand–receptor interactions involving T/NK cells and malignant cells, while large spatial analyses of high-grade serous tubo-ovarian cancer demonstrate that tumor-cell states and local tissue architecture predict lymphocyte infiltration and immune evasion [14,15,37,38,39].

### 5.1. IL-6 as a Central Regulatory Hub

Among the numerous cytokines identified within ovarian cancer ascites, IL-6 occupies a particularly relevant position. Accumulating evidence indicates that IL-6 contributes to multiple hallmarks of ovarian cancer progression, including tumor proliferation, angiogenesis, thrombopoiesis, cachexia, immune dysregulation, cancer-related anemia, and resistance to therapy [40]. Through activation of the Janus kinase/signal transducer and activator of transcription 3 (JAK/STAT3) pathway, IL-6 orchestrates extensive transcriptional programs affecting both malignant and non-malignant cellular populations [41].

The centrality of IL-6 is especially relevant because many of its biological effects simultaneously involve immune and metabolic regulation. Persistent STAT3 activation influences macrophage polarization, promotes tumor survival, facilitates angiogenesis, and contributes to immune dysfunction [41,42,43]. Consequently, IL-6 may be viewed not simply as an inflammatory cytokine but as a regulatory mediator linking inflammation, metabolism, iron handling, anemia, and immune competence [44].

### 5.2. Tumor-Associated Macrophages and Metabolic Reprogramming

Macrophages represent one of the most abundant and functionally diverse immune populations within ovarian cancer ascites. Earlier studies frequently described tumor-associated macrophages using an M1/M2 framework, which remains useful for interpreting selected functional observations but does not capture the full diversity revealed by contemporary single-cell and spatial analyses. Current data identify multiple macrophage states with distinct inflammatory, antigen-presenting, lipid-metabolic, iron-handling, angiogenic, and immunosuppressive programs. Their abundance and functional state vary with anatomical site, treatment context, molecular background, and ovarian cancer histotype. Thus, macrophage biology in ovarian cancer is better considered as a continuum of context-dependent states rather than a fixed binary polarization. Recent ovarian cancer studies further identify ascites-enriched lipid-loaded PLIN2-high macrophages and HRD-associated macrophage states with distinct spatial distributions, illustrating why a fixed M1/M2 classification is insufficient [45,46].

Glycolysis, oxidative phosphorylation, lipid metabolism, and iron handling collectively influence macrophage behavior and contribute to the formation of immunosuppressive microenvironments [28,29,30,31]. Several of these concepts were biologically foreshadowed by earlier investigations of ovarian cancer ascites. Alterations in macrophage-associated inflammatory pathways, cytokine production, and iron metabolism suggested that metabolic reprogramming was already an integral component of tumor–host interactions [28,29,30,31].

### 5.3. Iron Metabolism and Ferro-Metabolic Signaling

Iron metabolism has emerged as an important determinant of both tumor biology and immune regulation. Macrophages play a central role in iron recycling and storage, and disturbances in iron trafficking may influence oxidative stress, cytokine production, mitochondrial function, cellular proliferation, and cancer-related anemia [22]. Studies investigating ovarian cancer-associated inflammation demonstrated that alterations in iron homeostasis are closely linked to systemic inflammation, nutritional status, anemia, and disease progression [23].

Contemporary evidence supports a role for iron availability and ferroptosis susceptibility in ovarian cancer biology, but the level of evidence differs across compartments. Ovarian cancer-specific experimental studies demonstrate that tumor-cell ferroptosis sensitivity can be modified by iron availability and lipid/redox pathways. By contrast, direct demonstration that ferroptotic programs within human malignant ascites determine macrophage states or clinical immune escape remains limited. Accordingly, the proposed connection among ascitic iron handling, macrophage plasticity, and ferroptosis should be regarded as biologically plausible and hypothesis-generating rather than as an established mechanism in patients [24,25,47,48].

### 5.4. Adipocytes, Leptin, and Metabolic Crosstalk

The peritoneal cavity provides a unique biological context characterized by intimate interactions between ovarian cancer cells and adipose tissue. Adipocytes serve not only as energy reservoirs but also as active endocrine cells capable of producing adipokines, cytokines, and metabolic mediators [33,34,35]. Leptin, in particular, has attracted attention because of its involvement in inflammation, angiogenesis, energy homeostasis, and immune regulation [36]. The interaction between adipocytes, macrophages, and tumor cells contributes to a complex metabolic network that supports tumor growth and facilitates adaptation to environmental stress. Clinical evidence linking IL-6 and leptin to energy-metabolic alterations in advanced ovarian cancer further supports the relevance of this axis [36]. Such crosstalk reinforces the concept that ovarian cancer progression is driven not only by genetic alterations but also by dynamic interactions within an integrated immunometabolic ecosystem.

## 6. From Inflammation to Immunometabolism and Exhaustion-like Immune Dysfunction

The evolution of ovarian cancer biology over the last three decades has progressively transformed our understanding of inflammation. Initially viewed primarily as a host response to malignancy, inflammation is now recognized as a central biological driver capable of shaping tumor progression, immune competence, and metabolic adaptation [8]. In ovarian cancer, this transition from inflammation-centered models toward immunometabolic paradigms appears particularly evident. Cancer-associated cachexia illustrates this evolution. Historically considered a consequence of advanced disease, cachexia is now understood as a systemic syndrome driven by coordinated interactions among cytokines, immune cells, neuroendocrine pathways, and metabolic regulators [49]. IL-6, TNF-α, leptin dysregulation, and macrophage activation collectively contribute to profound alterations in protein turnover, lipid metabolism, mitochondrial function, and energy expenditure [50]. These same mediators simultaneously influence immune-cell function, thereby creating an integrated biological network linking inflammation and immune dysfunction [50].

The historical TALM findings should be interpreted as evidence of dysfunctional immune activation, not as a retrospective molecular diagnosis of T-cell exhaustion. Reduced proliferative responses, G0/G1 accumulation, altered Fas/CD25 signaling, and cytokine release demonstrated that immune cells could remain activated yet functionally ineffective within malignant effusions. These observations overlap phenomenologically with selected features seen in chronically stimulated T cells, but they did not assess the transcriptional, epigenetic, clonotypic, inhibitory-receptor, or differentiation programs that now define exhausted T-cell states. Contemporary single-cell and spatial studies in ovarian cancer can therefore be used to connect these early functional observations to directly characterized T-cell states, while preserving the distinction between historical evidence and modern mechanistic interpretation [19,20,21,37]. Modern ovarian cancer studies now directly characterize spatially confined exhausted T-cell states and their myeloid context, particularly after chemotherapy, providing the contemporary molecular evidence that was unavailable in the original TALM studies [51].

**Figure 2:**
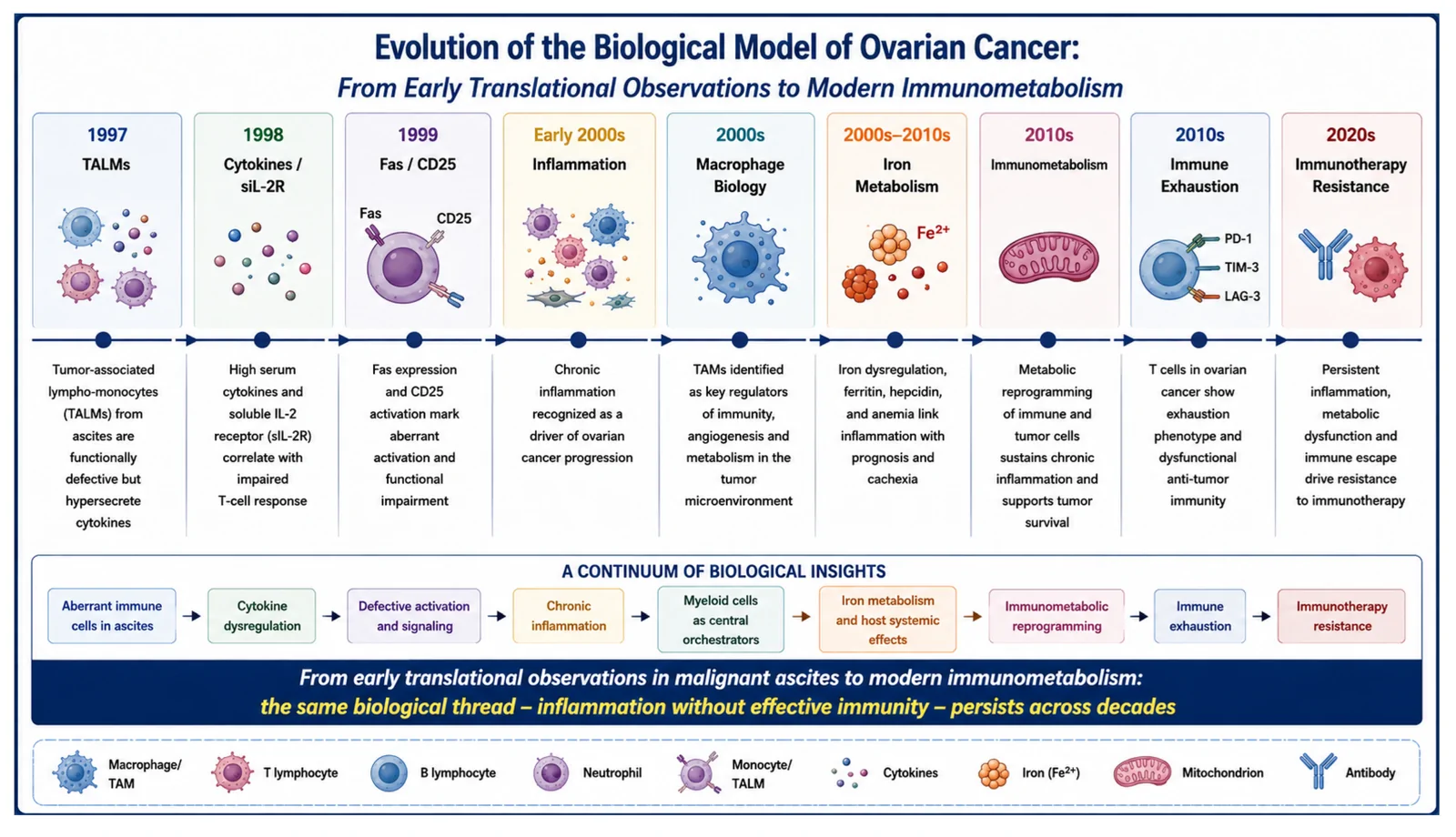
Conceptual continuum linking early translational observations in ovarian cancer ascites to the modern immunometabolic paradigm. The model emphasizes that chronic inflammatory activation and immune functional impairment were reported in early TALM studies and may now be interpreted through exhaustion-like immune dysfunction, macrophage polarization, and metabolic reprogramming.

The interpretation proposed here does not imply that early investigators identified immune exhaustion in its modern molecular definition. Rather, it highlights how careful translational observations recognized fundamental biological patterns whose significance became clearer after later advances in immunology and metabolism [52,53]. In this sense, ovarian cancer ascites may be considered an early functional model of inflammation associated with ineffective immunity. This framework also intersects with previous concepts linking chronic inflammation, regulatory T-cell (Treg) biology, macrophage activation, oxidative stress, IL-6 production, altered iron metabolism, and metabolic dysfunction. Persistent inflammatory activation may progressively impair T-cell competence and promote immune suppression [19,20,21]. Thus, chronic inflammation and immune dysfunction should not be considered opposing processes but interconnected manifestations of the same pathological ecosystem (Figure 2).

## 7. Implications for Immunotherapy Resistance

Clinical experience with immune-checkpoint inhibition in ovarian cancer illustrates the distinction between immune-cell presence and therapeutically effective immunity. Early single-agent PD-1/PD-L1 studies produced reproducible but generally low response rates, and several large randomized trials incorporating checkpoint blockade into frontline or recurrent-disease regimens failed to improve outcomes in unselected populations [54,55,56,57,58]. These findings argue against checkpoint signaling alone as a sufficient explanation for immune dysfunction. Importantly, the 2026 ENGOT-ov65/KEYNOTE-B96 trial demonstrated that pembrolizumab combined with weekly paclitaxel, with or without bevacizumab, improved clinical outcomes in platinum-resistant recurrent disease, with a clinically relevant effect in the PD-L1 CPS ≥ 1 population [59]. Immune resistance in ovarian cancer should therefore not be regarded as absolute: efficacy appears dependent on biological context, therapeutic combination, and patient selection. Histotype composition must also be considered when interpreting aggregate trial results because different epithelial ovarian cancer subtypes have distinct immune microenvironments [54,55,56,57,58,59,60,61]. Table 2 shows the clinical trials that evaluated immunotherapy in the treatment of patients with ovarian cancer, both as monotherapy and in combination with chemotherapy.

At present, immune-checkpoint inhibition has the strongest direct clinical evidence among the therapeutic concepts discussed here, although benefit is context- and biomarker-dependent. Macrophage reprogramming, IL-6/JAK/STAT3 modulation, ferroptosis targeting, and manipulation of iron or lipid metabolism remain biologically compelling but are not established standards for ovarian cancer ascites. Their inclusion in this review is intended to define testable translational hypotheses and biomarker strategies rather than to recommend clinical use outside appropriate trials.

Persistent IL-6 signaling may represent one driver of this process. Chronic activation of the JAK/STAT3 pathway promotes tumor survival while simultaneously impairing effective antitumor immunity [40,41,42,43,44]. Sustained inflammatory stimulation may further contribute to exhaustion-like phenotypes characterized by reduced proliferative capacity and diminished effector function. TAMs constitute another major source of resistance through cytokine production, angiogenic signaling, extracellular matrix remodeling, metabolic regulation, and modulation of nutrient availability, iron metabolism, and mitochondrial function [28,29,30,32].

Metabolic competition represents an additional layer of complexity. Tumor cells, activated macrophages, and dysfunctional lymphocytes coexist within an environment characterized by limited nutrient availability and altered metabolic fluxes [50]. Glucose deprivation, amino acid depletion, lactate accumulation, oxidative stress, and mitochondrial dysfunction may contribute to progressive immune impairment [32]. Hypoxia further amplifies these mechanisms by promoting angiogenesis, metabolic adaptation, and immune suppression. The resulting biological landscape favors tumor persistence despite the presence of immune-cell infiltration [62,63,64]. Viewed through this integrated framework, ovarian cancer should not be regarded simply as immunologically cold. Rather, it may represent a biologically active example of inflammation-associated immune dysfunction. Strategies based solely on immune checkpoint inhibition may therefore be insufficient because they target only one component of a broader inflammatory–immunometabolic network [65].

## 8. Future Therapeutic Perspectives: Beyond Tumor-Centered Therapy

The therapeutic evolution of ovarian cancer has been largely driven by tumor-centered strategies aimed at directly targeting malignant cells. Cytoreductive surgery, chemotherapy, anti-angiogenic agents, PARP inhibitors, antibody–drug conjugates, and platinum-based therapy have all contributed to improved outcomes [1,2,66,67]. Nevertheless, durable disease control remains elusive for many patients. The immunometabolic model proposed in this review suggests that future progress may depend on a broader therapeutic vision in which both tumor cells and their biological ecosystem become treatment targets [66]. Table 3 shows a summary of the biological axes involved in generalized inflammation, proposed as potential targets for translational medicine, discussed below.

### 8.1. Targeting the IL-6/STAT3 Axis

Among inflammatory pathways, IL-6 occupies an attractive position because of its central role in connecting inflammation, immune dysfunction, thrombopoiesis, cachexia, angiogenesis, anemia, and tumor progression [26,36]. Persistent activation of STAT3 signaling has been implicated in multiple mechanisms of therapeutic resistance. Consequently, inhibition of the IL-6/STAT3 pathway may provide benefits extending beyond direct tumor effects by simultaneously modulating immune and metabolic compartments [41,42,43]. Future studies should evaluate whether inflammatory biomarkers can identify subsets of patients most likely to benefit from cytokine-directed interventions.

### 8.2. Macrophage Reprogramming

TAMs have emerged as critical orchestrators of the ovarian cancer microenvironment [23]. Their contribution extends across disease progression, immune suppression, angiogenesis, extracellular matrix remodeling, iron regulation, anemia, and metabolic adaptation [28,29,30,31]. Rather than eliminating macrophages, emerging strategies increasingly focus on functional reprogramming. Converting tumor-promoting macrophages into immune-supportive populations could enhance antitumor immunity while disrupting mechanisms of tumor progression [29]. The ascitic microenvironment may represent an attractive setting for evaluating macrophage-directed interventions because of the abundance and accessibility of these cellular populations [33,34,35].

### 8.3. Iron Metabolism, Ferroptosis, and Macrophage Plasticity

The growing recognition of iron metabolism as a regulator of tumor biology opens additional therapeutic opportunities. Iron availability influences oxidative stress, mitochondrial activity, macrophage function, anemia, and susceptibility to ferroptosis [22,23,24,25,27]. Because ovarian cancer frequently develops within an inflammatory environment characterized by altered iron handling, modulation of iron-metabolic pathways may represent a rational therapeutic vulnerability [68].

Ferroptosis should not be considered solely as a direct tumor-cell killing mechanism. Ferroptotic stress may also influence the tumor microenvironment through lipid peroxidation products, oxidized phospholipids, damage-associated molecular patterns, macrophage recruitment, and functional polarization [47,48,69,70]. In ovarian cancer ascites and metastatic niches, macrophages are exposed to oxidative stress, iron flux alterations, and lipid-rich microenvironments, making the interaction between ferroptosis and macrophage plasticity biologically plausible [71]. However, the immunological consequences of ferroptosis are context dependent. Extensive ferroptosis may support immunogenic signaling, whereas incomplete or sublethal ferroptotic programs may favor adaptive responses that support tumor persistence. Effective ferroptosis-based strategies will therefore likely require simultaneous modulation of macrophage reprogramming and immune suppression pathways to convert oxidative stress into productive antitumor immunity rather than tumor adaptation [71,72,73].

### 8.4. Metabolic Therapy

Cancer metabolism has traditionally focused on tumor-cell energetics. However, contemporary evidence increasingly demonstrates that metabolism regulates the behavior of virtually every component of the tumor microenvironment [18,63,64,65]. T lymphocytes, macrophages, fibroblasts, adipocytes, and endothelial cells all undergo metabolic adaptations that influence therapeutic response [66]. Interventions targeting glucose utilization, mitochondrial function, lipid metabolism, amino acid pathways, oxidative stress, or inflammation may therefore exert effects extending beyond direct tumor inhibition [50,66]. Within this framework, metabolic therapy may also function as immune therapy and inflammatory therapy.

### 8.5. PARP Inhibition as a Metabolic and Immunomodulatory Intervention

**Figure 3:**
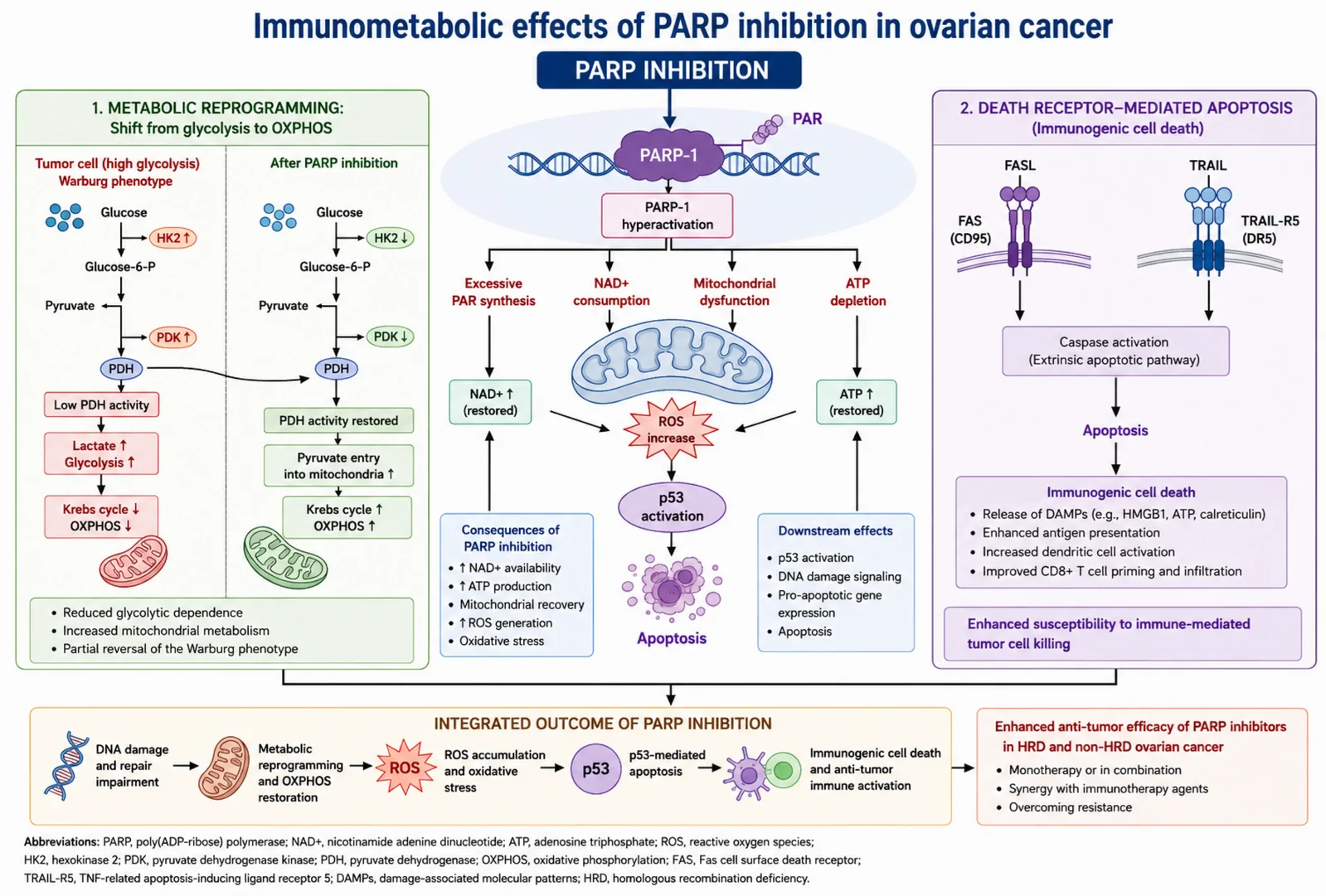
Immunometabolic and immunomodulatory effects of PARP inhibition in ovarian cancer. PARP inhibition may influence tumor-cell metabolism, redox balance, mitochondrial function, DNA-damage responses, and immunogenic signaling in addition to its established effects on DNA repair. These mechanisms are supported predominantly by experimental evidence and are presented as potential modifiers of the ovarian cancer immunometabolic ecosystem rather than as established mechanisms in malignant ascites.

PARP inhibitors are established primarily through synthetic-lethality and DNA-repair mechanisms, particularly in homologous recombination-deficient tumors. Experimental literature also links PARP activity to NAD+ consumption, mitochondrial function, redox balance, innate immune signaling, and interactions with checkpoint blockade. However, these metabolic and immunomodulatory effects are not yet sufficiently demonstrated in human ovarian cancer ascites to constitute a central pillar of the present framework. PARP inhibition is therefore considered here as a potentially relevant modifier of the immunometabolic ecosystem rather than as evidence for the model itself; mechanistic claims should be supported by original experimental studies rather than commentary alone [74]. Figure 3 shows the immunometabolic and immunomodulatory mechanisms induced by PARP inhibitors in ovarian cancer.

### 8.6. Combinatorial Immunotherapy

The modest activity of single-agent checkpoint inhibitors suggests that ovarian cancer may require combinatorial therapeutic strategies [5]. Future regimens may integrate immune checkpoint inhibition, IL-6/STAT3 targeting, macrophage reprogramming, anti-angiogenic therapy, ferroptosis-based approaches, metabolic modulation, antibody–drug conjugates, PARP inhibitors, and platinum-based chemotherapy. The challenge will not simply be identifying effective combinations but selecting biologically appropriate combinations for specific immunometabolic phenotypes [3,65,66].

### 8.7. Precision Immunometabolism

The next generation of ovarian cancer treatment may depend on the development of precision immunometabolism. Rather than classifying patients exclusively according to histology, stage, or genomic alterations, future models may incorporate inflammatory signatures, metabolic profiles, cytokine networks, macrophage phenotypes, ascitic biomarkers, iron-related parameters, and sarcopenia/cachexia indicators. Candidate features of inflammation without effective immunity may include high inflammatory cytokine activity, abundant TAMs, impaired lymphocyte responsiveness, altered iron homeostasis, anemia of inflammation, cachexia or sarcopenia, hypoxia, lactate accumulation, mitochondrial stress, and ascites enriched in suppressive immune populations. Such approaches could allow therapeutic strategies to be tailored according to the dominant biological drivers operating within each patient’s tumor ecosystem [14,15,37,65,75].

## 9. Future Perspectives

The convergence of immunology, metabolism, inflammation, and systems biology is progressively transforming our understanding of ovarian cancer. Several priorities appear particularly relevant for future research. First, the identification of reproducible inflammatory phenotypes may improve biological stratification and facilitate personalized therapeutic approaches [75,76]. Second, comprehensive profiling of malignant ascites should be further developed as a source of biomarkers capable of integrating immune, metabolic, and inflammatory information [33,34,35]. Third, advances in spatial biology and single-cell technologies are providing increasingly direct insight into cellular interactions occurring within the ovarian cancer microenvironment [14,15,37]. Fourth, ferro-metabolic pathways, macrophage heterogeneity, and cytokine-network architecture require further investigation to clarify their contribution to disease progression and therapeutic resistance [69,71]. Finally, integrative computational approaches may facilitate the interpretation of increasingly complex biological datasets, allowing dynamic modeling of tumor–host interactions and identification of previously unrecognized therapeutic vulnerabilities. Collectively, these developments support a transition from reductionist models toward ecosystem-based interpretations of ovarian cancer biology.

## 10. Limitations of the Proposed Framework

Several limitations should be acknowledged. First, the historical studies discussed in this review were performed before the availability of contemporary single-cell, spatial, transcriptomic, and epigenetic technologies; therefore, their findings cannot be directly equated with modern molecular definitions of immune exhaustion, macrophage polarization, or ferroptosis-related immune regulation. Second, the concept of inflammation without effective immunity remains an integrative interpretive framework rather than a validated biomarker-defined clinical state. Third, several mechanistic links among IL-6 signaling, iron metabolism, ferroptosis, macrophage plasticity, adipocyte-derived signaling, PARP-related metabolic effects, and immunotherapy resistance remain incompletely established in ovarian cancer. Fourth, the therapeutic implications discussed here require prospective validation in biomarker-selected clinical studies. These limitations do not weaken the biological relevance of the proposed framework, but they indicate that it should be interpreted as a translational hypothesis requiring further mechanistic and clinical validation.

## 11. Conclusions

The history of ovarian cancer research provides an example of biological observations preceding the conceptual frameworks later used to interpret them. Studies performed on malignant effusions and tumor-associated lymphomonocytes in the 1990s demonstrated impaired proliferative responses, altered activation signaling, and substantial cytokine release despite the presence of immune cells. These findings constitute direct historical evidence of dysfunctional immune activation; they should not be equated with modern molecular definitions of T-cell exhaustion, macrophage states, or ferroptosis [11,12,13].

## Figures and Tables

**Table 1:** Historical translational observations and contemporary immunometabolic interpretation.

Historical Observation	Biological Compartment	Contemporary Interpretation	Interpretive Caution	Representative References
Reduced TALM proliferative response	Malignant effusions/TALMs	Functional immune impairment despite local immune-cell presence	Not equivalent to a modern molecular diagnosis of T-cell exhaustion	[11,12,13,19,20,21]
High IL-6, TNF-α, IL-10, IFN-γ, and soluble IL-2 receptor	Ascites and systemic inflammatory milieu	Persistent inflammatory activation associated with ineffective immunity	Cytokine elevation alone does not prove causality	[10,11,12,13]
Altered Fas/CD25 signaling	Tumor-associated lymphomonocytes	Activation-marker expression coexisting with defective immune function	Requires modern phenotyping for mechanistic validation	[12]
Inflammation, anemia, and altered iron handling	Systemic host response/macrophage-related biology	Link between inflammation, iron metabolism, and immune-metabolic dysregulation	Ferroptosis-related interpretation is contemporary and partly inferential	[22,23,24,25,26,27]
Macrophage-associated inflammatory pathways	Ascites and tumor microenvironment	Potential interface between cytokine release, immune suppression, and metabolic adaptation	Historical studies did not define current TAM subsets at single-cell resolution	[23,28,29,30,31,32]
IL-6/leptin and energy-metabolic alterations	Host metabolic response and adipocyte-related signaling	Integration of inflammation, cachexia, adipokines, and metabolic stress	Clinical biomarker validation remains required	[26,33,34,35,36]

For clarity, the proposed immunometabolic ecosystem is organized around five interacting axes: (1) cytokine-driven chronic inflammation; (2) heterogeneous tumor-associated macrophage states; (3) iron/redox regulation and ferroptosis-related susceptibility; (4) adipocyte–tumor metabolic crosstalk and systemic cachexia; and (5) dysfunctional or exhaustion-like T-cell states. These axes are not expected to be equally expressed across histotypes or anatomical compartments. Each of the historical observations reported in the table, as well as the five integrating axes, will be discussed in greater detail in the text below, with the relevant references provided.

**Table 2:** Representative immune-checkpoint inhibitor trials in epithelial ovarian cancer and their relevance to the inflammation-without-effective-immunity framework.

Trial	Setting/n	Treatment	ORR/Main Clinical Result	Biomarker Finding
KEYNOTE-100 [55]	Recurrent OC, n = 376	Pembrolizumab	ORR 7.4–9.9%; CPS < 1, 4.1%, CPS ≥ 10, 10%	Modest increase in response with increasing PD-L1 CPS
NRG-GY003 [56]	Recurrent/persistent, n = 100	Nivolumab vs. nivolumab + ipilimumab	ORR 12.2% vs. 31.4%	Dual checkpoint blockade produced greater activity than monotherapy, but no robust predictive biomarker was established
JAVELIN Ovarian 200 [57]	Platinum-resistant/refractory, n = 566	Avelumab ± PLD vs. PLD	ORR approximately 4% with avelumab, 13% with combination therapy, and 4% with PLD; no significant improvement in PFS or OS	PD-L1 expression and CD8^+^ T-cell infiltration were insufficient as predictive biomarkers
IMagyn050 [60]	Newly diagnosed stage III–IV, n ≈ 1300	Atezolizumab + bevacizumab + chemotherapy	No significant improvement in PFS in the overall population	PD-L1 positivity did not identify a convincing population with clinically meaningful benefit
ATALANTE/ENGOT-ov29 [61]	Platinum-sensitive recurrence, n = 614	Atezolizumab + bevacizumab + platinum chemotherapy	ORR 62% vs. 66%; no significant improvement in PFS	PD-L1 expression was not predictive of treatment benefit
NINJA [58]	Platinum-resistant, n = 316	Nivolumab vs. GEM/PLD	ORR 7.6% vs. 13.2%; nivolumab did not improve OS and was associated with inferior PFS	PD-1 monotherapy was insufficient; no clinically useful predictive biomarker was established
KEYNOTE-B96/ENGOT-ov65 [59]	Platinum-resistant recurrence, n = 643	Pembrolizumab + weekly paclitaxel ± bevacizumab	ORR 53.0% vs. 46.6% in CPS ≥ 1; OS 18.2 vs. 14.0 months	PD-L1 CPS ≥ 1 identifies the clinically relevant population

OC, ovarian cancer; n, number of patients in the study; ORR, objective response rate; OS, overall survival; PFS, progression-free survival; ICI, immune checkpoint inhibitor; CPS, PD-L1 combined positive score; PD-1, programmed cell death protein 1; PD-L1, programmed death-ligand 1; PLD, pegylated liposomal doxorubicin; GEM, gemcitabine.

**Table 3:** Candidate biological axes of the inflammation-without-effective-immunity model.

Biological Axis	Mechanistic Role Within the Model	Potential Translational Implication	Current Limitation
IL-6/STAT3	Links cytokine activation, tumor survival, angiogenesis, anemia, cachexia, and immune dysfunction	Biomarker-driven cytokine-directed strategies	Requires prospective validation in selected patients
TAM biology	Coordinates immune suppression, angiogenesis, extracellular matrix remodeling, iron handling, and metabolic adaptation	Macrophage reprogramming rather than nonspecific depletion	TAM heterogeneity is incompletely captured by historical studies
Iron metabolism/ferroptosis	Connects inflammation, oxidative stress, macrophage plasticity, anemia, and ferroptotic susceptibility	Combination of ferroptosis modulation with immune and macrophage-directed strategies	Context-dependent effects may be tumor-suppressive or tumor-promoting
Adipocyte-leptin-metabolic crosstalk	Supports energy adaptation, inflammation, angiogenesis, and host metabolic dysregulation	Integration of metabolic and inflammatory biomarkers	Needs stronger clinical biomarker standardization
PARP/NAD+/redox biology	May influence DNA repair vulnerability, mitochondrial function, oxidative stress, and metabolic reprogramming	Rationale for combinations with immunotherapy and metabolic targeting	Metabolic effects remain complementary to the established DNA repair rationale
Precision immunometabolism	Integrates cytokines, immune cells, metabolism, iron parameters, ascites biomarkers, and cachexia indicators	Patient stratification beyond histology and genomics alone	No validated clinical classification is yet available

## Data Availability

No new data were created or analyzed in this study. Data sharing is not applicable to this article.
